# Spontaneous Umbilical Cord Hematoma

**DOI:** 10.7759/cureus.13048

**Published:** 2021-02-01

**Authors:** Pratichhya Khatiwada, Mohammed Alsabri, Salome Wiredu, Viswanathan Kusum, Vohra Kiran

**Affiliations:** 1 Department of Pediatrics, Brookdale University Hospital Medical Center, Brooklyn, USA; 2 Department of Pediatrics, Division of Pediatric Hematology/Oncology, Brookdale University Hospital Medical Center, Brooklyn, USA; 3 Department of Neonatology, Brookdale University Hospital Medical Center, Brooklyn, USA

**Keywords:** umbilical cord, hematoma, complications

## Abstract

Spontaneous umbilical cord hematoma is a rare complication with a usually benign course but is potentially fatal without vigilant and timely medical intervention.

We present the case of a 23-year-old primigravida mother who presented in labor. She was placed on continuous fetal heart rate monitoring, which showed two episodes of fetal heart rate tracing of the category II variety. The labor was induced with oxytocin, and the ammonitic membrane was incised artificially. The baby was male, term at 38 weeks, with an appropriate weight, length, and head circumference. There was no gross anomaly or dysmorphic features; the APGAR (Appearance, Pulse, Grimace, Activity, and Respiration) score of the baby was 9 and 9 at the first and fifth minutes, respectively. A 4.5 cm hematoma was discovered on the umbilical cord immediately following delivery. He was admitted to the regular nursery for routine newborn care and was discharged home in stable condition.

Spontaneous umbilical cord hematoma is usually due to the rupture of the umbilical vein. Mostly, the umbilical cord hematoma occurs spontaneously and often follows a benign course, however, in some cases, the perinatal loss secondary to umbilical cord hematoma could very high, especially if associated with abnormal fetal heart rate tracing. Because of the potential for fatality inherent in this condition and to understand the clinical manifestations, risk factors, and eventual course of spontaneous cord hematoma, we present this case to help fellow pediatricians reduce morbidity and mortality associated with it.

## Introduction

Umbilical cord hematoma is defined as the extravasation of blood, mainly venous, in the Warton's jelly that covers the umbilical vessels [1]. In about 10% of cases, it could be due to arterial bleeding. It is considered rare, accounting for about one in 5500-11000 living births [2-3]. Mostly, an umbilical cord hematoma occurs spontaneously and the exact cause is often not detected [3]. However, the cord anomaly (a short cord, traction, knot, prolapse), iatrogenic (amniocentesis, instrumentation), infection, coagulation disorder, and post maturity should be considered as the potential causes [2,4-5]. Nevertheless, most hematomas are difficult to be diagnosed by perinatal ultrasonography and when discovered in utero, it requires higher clinical attention as the rate of fetal demise is high [2]. The hematomas could be asymptomatic when they occur in the peripartum period or could present with only abnormal fetal heart rate tracing [3]. The perinatal loss for umbilical cord hematoma could be as high as 50% [3-4]. On the other hand, if there is no abnormal tracing of the fetal heart rate, it could be found incidentally after birth and may follow a benign course [3].

## Case presentation

**Figure 1:**
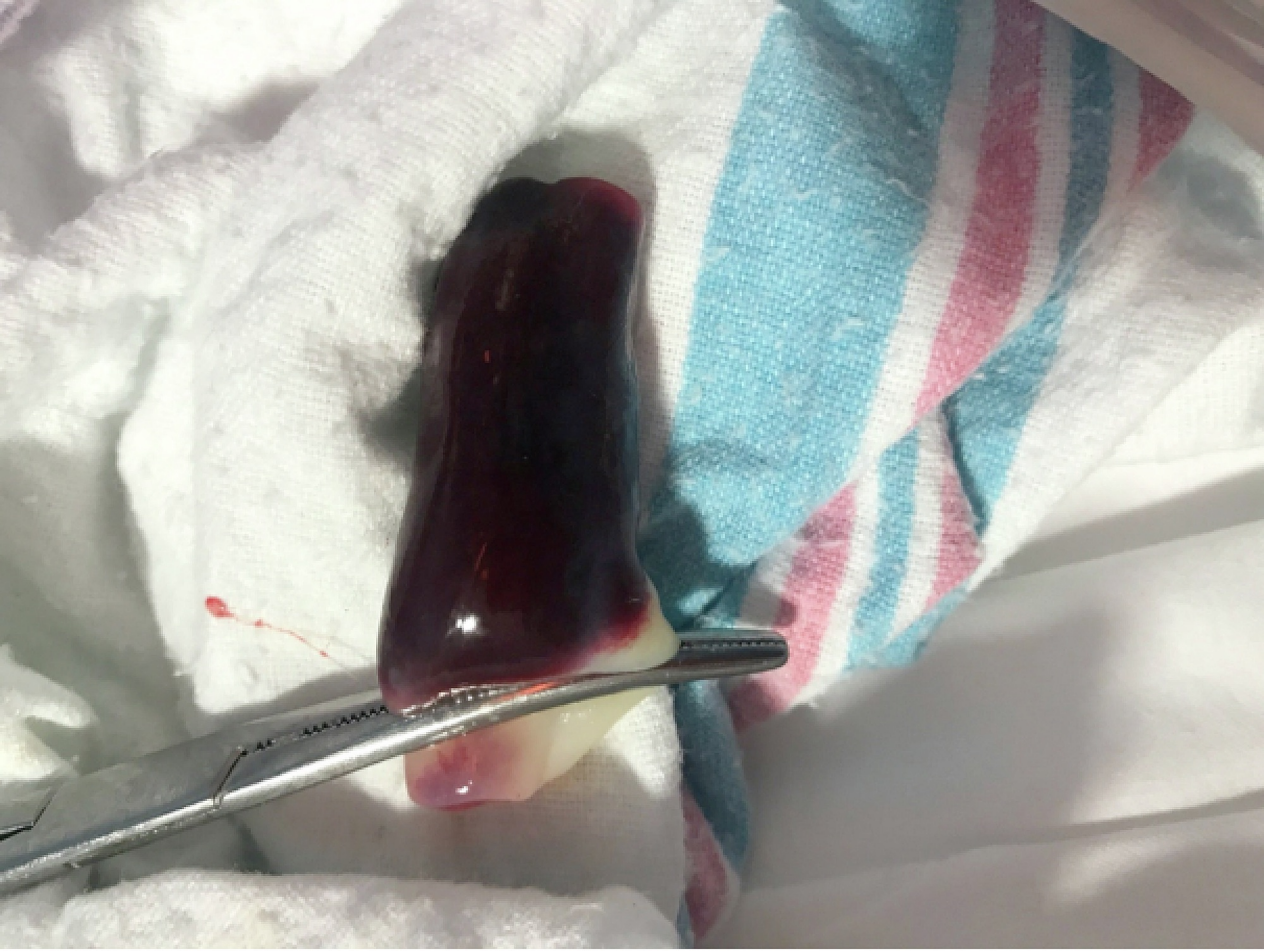
Umbilical cord hematoma soon after birth

**Figure 2:**
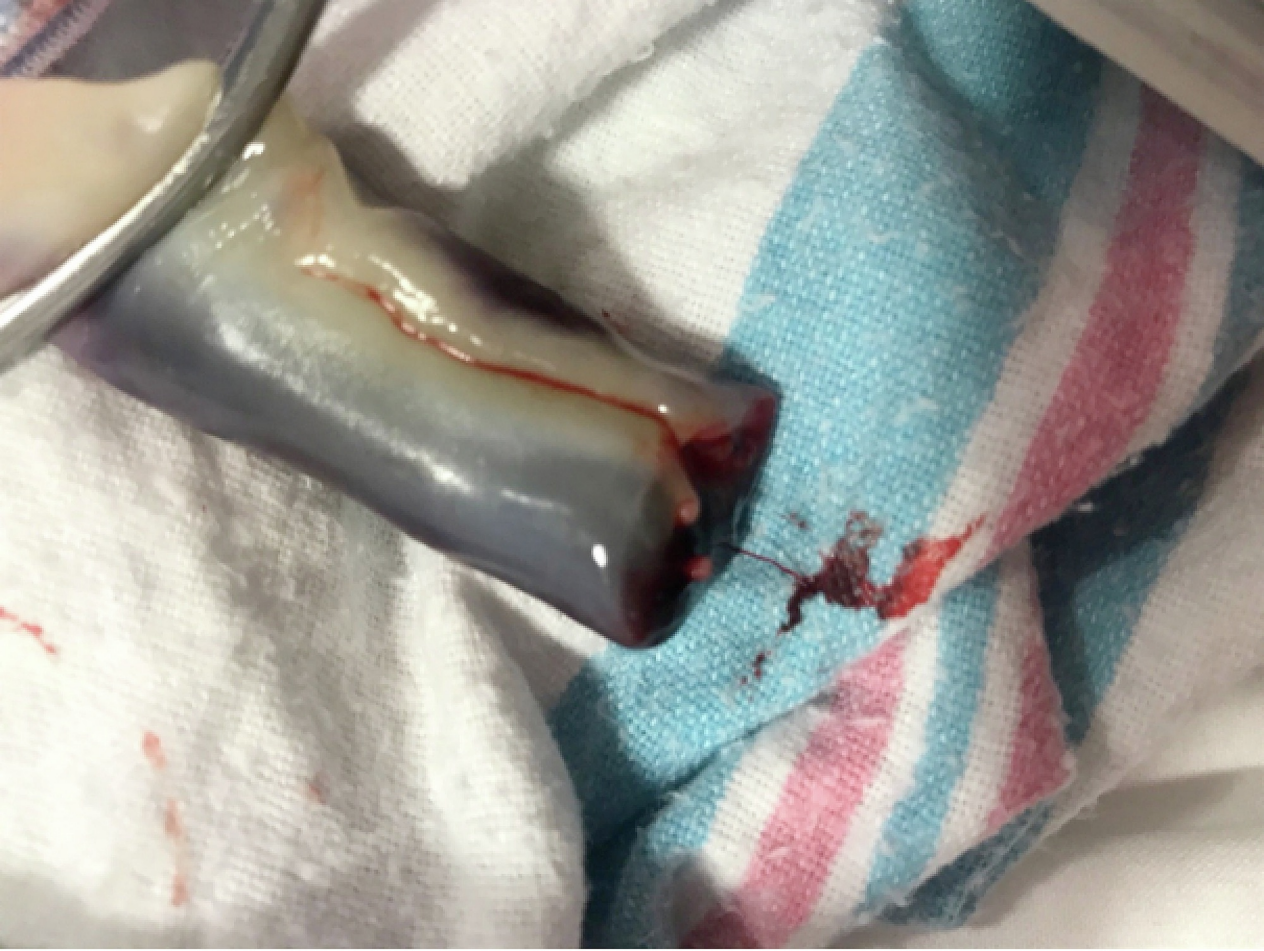
Umbilical cord hematoma soon after birth

**Figure 3:**
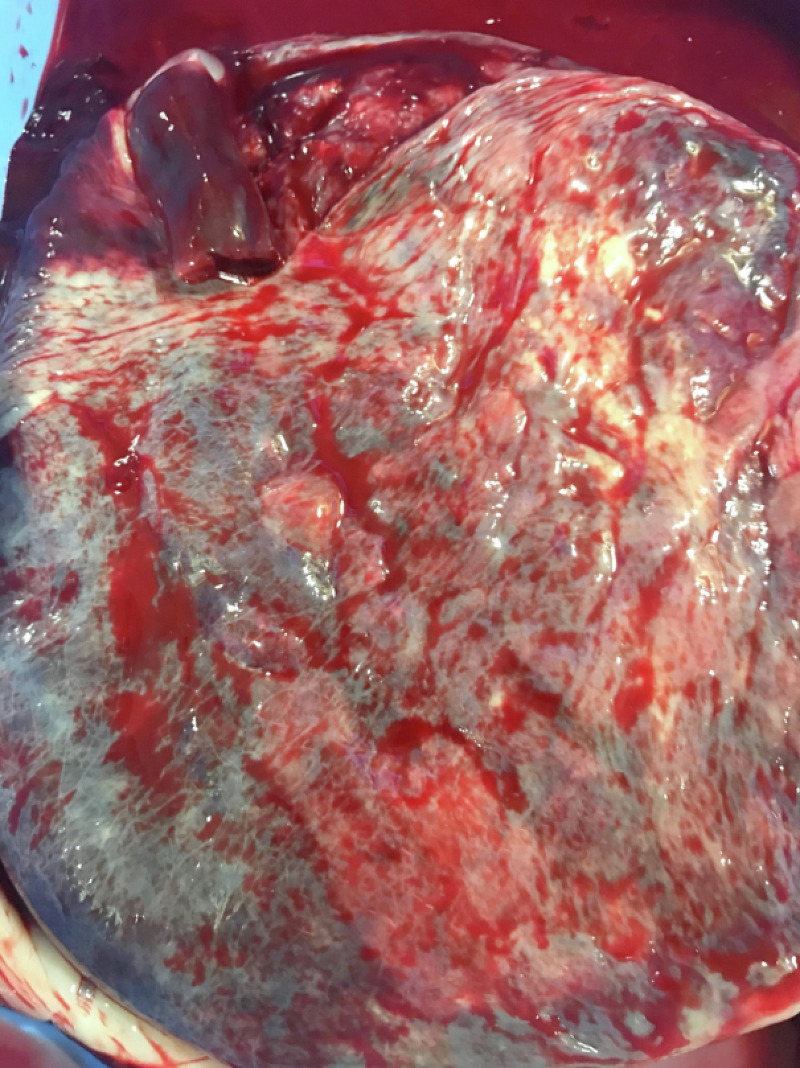
Placenta presenting with a reddish to purple swelling and measured about 4X 5 cm soon after birth

Our patient is a 23-year-old primigravida mother who presented to the labor and delivery department with abdominal pain. She had a history of cervical insufficiency with cervical cerclage placed in the third trimester and was taking daily progesterone. The cerclage was removed the week prior to the presentation. The antenatal labs were insignificant for gonorrhea, chlamydia, Guillain-Barré syndrome (GBS), human immunodeficiency syndrome (HIV), and Hepatitis B. She denied a history of hypertension, cigarette smoking, or consumption of any illicit substances. Her abdominal pain became progressive but her labor was delayed, requiring augmentation with oxytocin. Epidural anesthesia was given when her pain scale was of moderate intensity (5/10). Artificial rupture of the membrane was performed six hours prior to birth, which revealed clear amniotic fluid. The continuous fetal heart rate monitoring was placed and showed two episodes of fetal heart rate tracing of category II. The episodes resolved with maternal positioning, and supplemental oxygen to mother at 10 L/min. The APGAR (Appearance, Pulse, Grimace, Activity, and Respiration) score of the baby was 9 and 9 at the first and fifth minutes, respectively. The neonate was assessed by the pediatric team, and the umbilical cord was found to be stained black and red, with a size of 4x3 cm at the point of insertion into the fetal side. The cord was edematous and a cord hematoma was diagnosed (Figures 1-2). The baby was a full-term male with an appropriate weight, length, and head circumference. There were no gross anomaly or dysmorphic features. However, the placenta had clots measuring 4x5 cm (Figure 3). The heart rate was regular and no murmur was audible. There was no active bleeding, hematoma, or bruise from any other side.

**Figure 4:**
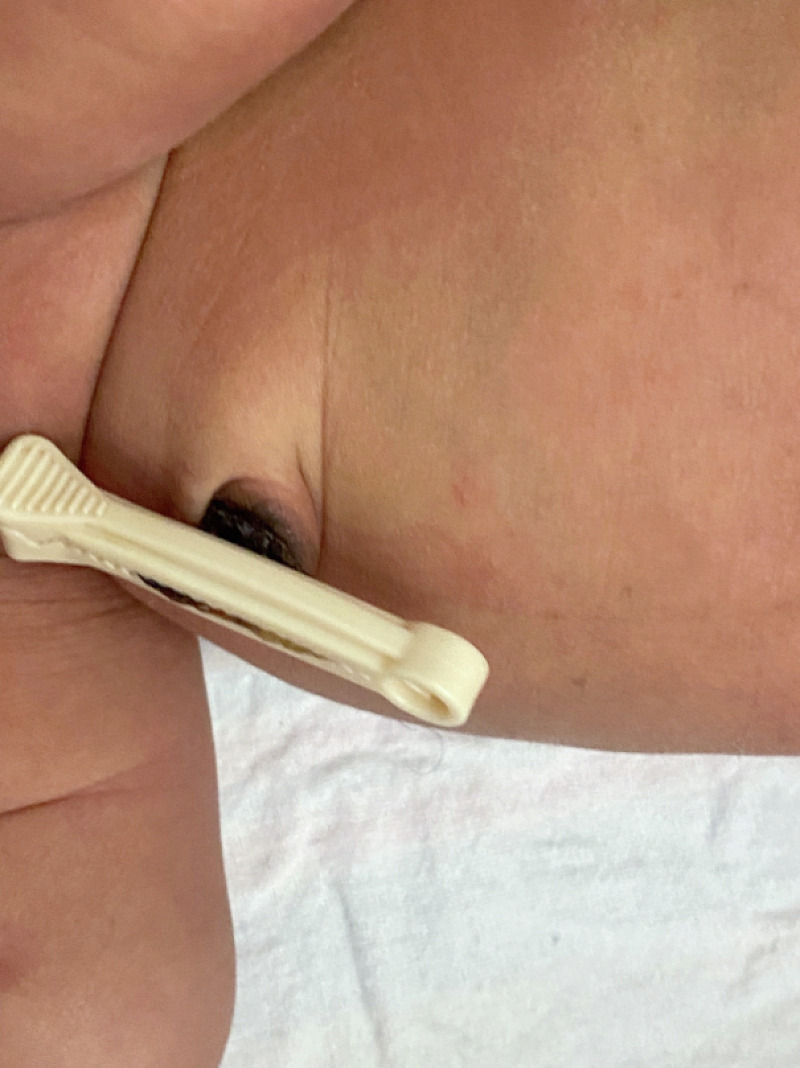
Umbilical cord after 36 hours of birth

He was admitted to a regular nursery for routine newborn care. He was fed breast milk and was vitally stable with no concern of bleeding from the umbilical hematoma. The bilirubin levels at the corresponding age in hours were insignificant. The umbilical cord was followed at 36 hours of life, which had dried (Figures 3-4). The hematoma had resolved without any complications like bleeding or infection. Mother and baby were both discharged within 48 hours of life with an appointment with a primary care doctor scheduled per routine follow-up protocol, which within our institution is two days. At the office of the primary medical doctor, the complete blood count and bilirubin level were repeated and the labs were insignificant, the umbilical cord was dry, and there was no evidence of hematoma. 

## Discussion

Clinical significance

Given that this is a rare complication (one in 5500-11000) with a usually benign course but potentially fatal, it is important to be able to perform a thorough examination of the umbilical cord in order to ascertain that it is indeed benign and does not fall into the category of a pathological course in the rare occasion where complications may be present [3-4]. As we mentioned above, in some, an umbilical hematoma can lead to pathological and even fatal outcomes, including perinatal asphyxia and stillbirth [6]. This means that its etiology ought to be considered and where intragenic causes are present, clinicians ought to work to try to limit the possible complications of procedures. It is also then of paramount importance to strengthen placental and cord examinations especially in case of unexplained stillbirth or fetal hypoxia. If the hematoma occurs, the peripartum could be asymptomatic and could present with only abnormal fetal heart rate tracing [3]. If abnormal heart rate tracing is present, an emergent C-section should be performed to save potential fetal loss.

In certain cases, the etiology of the hematoma can be a ruptured vein. The ruptured vein can be spontaneous, secondary to the alteration and inflammation of the vessel wall, or secondary to in-utero instrumentations from amniocentesis, fetal transfusions, and fetal diagnostic procedures [4-5].

The change in the architecture of the vessels leading to the hematoma is then thought to impede blood flow through the surrounding vessels, possibly resulting in fetal asphyxia and possible demise [3-4]. It is important to note that though the spontaneous rupture of umbilical vessels cannot be controlled, clinicians can control the secondary causes resulting in umbilical hematomas by exercising more caution with amniocentesis and other diagnostic fetal procedures in order to avoid vessel insult, possible rupture, and resulting hematoma. Ethically and from a medico-legal standpoint, clinicians ought to list umbilical hematomas as possible complications of amniocentesis and other diagnostic fetal procedures so as to give full and comprehensive informed consent in order to protect both the patient and themselves. In our case, it seemed that none of the earlier described etiological factors were involved except the cerclage, which was not invasive enough to cause a hematoma on the fetal side of the cord. In addition, no trauma occurred during labor, making it likely a spontaneous phenomenon.

## Conclusions

A spontaneous umbilical cord is a rare condition and usually occurs due to the rupture of the umbilical vein. Mostly, the umbilical cord hematoma occurs spontaneously and often follows a benign course; however, in some cases, the perinatal loss secondary to an umbilical cord hematoma could be as high as 50%.
